# Differential Regulation of Glucosylceramide Synthesis and Efflux by Golgi and Plasma Membrane Bound ABCC10

**DOI:** 10.3390/nu15020346

**Published:** 2023-01-10

**Authors:** Jahangir Iqbal, Abeer Al Otaibi, Sindiyan Alshaikh Mubarak, Ali Alqarni, Ahmed Bakillah

**Affiliations:** King Abdullah International Medical Research Center-Eastern Region, King Saud Bin Abdulaziz University for Health Sciences, King Abdulaziz Hospital, Ministry of National Guard-Health Affairs, Al Ahsa 31982, Saudi Arabia

**Keywords:** glucosylceramide, ceramide, sphingomyelin, sphingolipids, ABCC10

## Abstract

Glucosylceramide (GlcCer) synthesis by the enzyme glucosylceramide synthase (GCS) occurs on the cytosolic leaflet of the Golgi and is the first important step for the synthesis of complex glycosphingolipids (GSLs) that takes place inside the lumen. Apart from serving as a precursor for glycosylation, newly synthesized GlcCer is also transported to the plasma membrane and secreted onto HDL in the circulation. The mechanism by which GlcCer is transported to HDL remains unclear. Recently, we showed that ATP-binding cassette transporter protein C10 (ABCC10) plays an important role in the synthesis and efflux of GlcCer in Huh-7 cells. In this study, we found that treatment of Huh-7 cells with an ABCC10 inhibitor, sorafenib, decreased the synthesis and efflux of GlcCer. However, treatment of cells with cepharanthine reduced only the efflux, but not synthesis, of GlcCer. These results indicate that ABCC10 may regulate the synthesis and efflux of GlcCer differentially in liver cells.

## 1. Introduction

**Figure 1:**
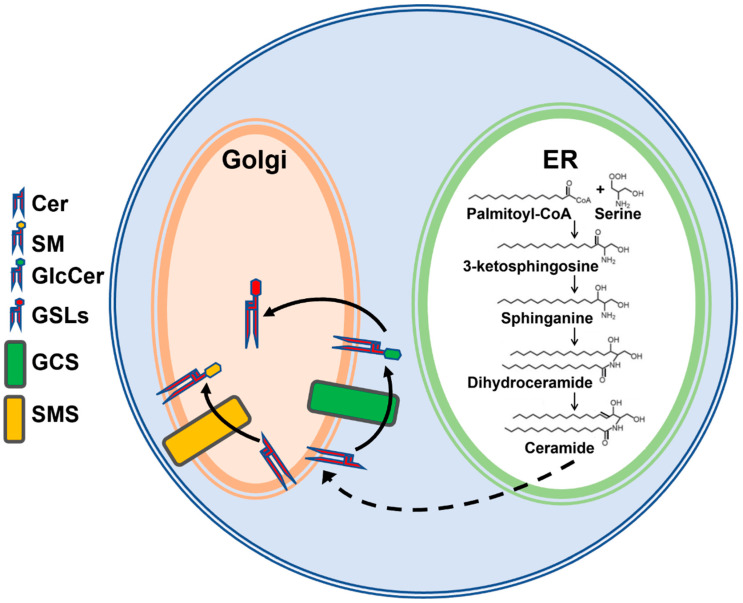
Simplified schematic representation of sphingolipid biosynthesis. De novo sphingolipid synthesis is initiated by the condensation of palmitoyl-CoA and serine in the endoplasmic reticulum (ER), leading to the synthesis of ceramide (Cer). Cer is delivered to the Golgi by transport proteins or vesicular transport for synthesis of sphingomyelin (SM) on the lumenal side of Golgi by sphingomyelin synthase (SMS) or glucosylceramide (GlcCer) on the cytosolic side of Golgi by glucosylceramide synthase (GCS). GlcCer is then translocated to the Golgi lumen for the biosynthesis of complex glycosphingolipids (GSLs).

Sphingolipids are important bioactive components of cells that play a critical role in several metabolic and hormonal pathways. Studies on sphingolipids have intensified in the past decade. Much is known about the synthesis and signaling activities of sphingolipids [1,2,3,4,5]. The control of sphingolipid synthesis is regulated by various enzymes [6,7,8], beginning at the cytosolic leaflet of the endoplasmic reticulum (ER) to generate ceramide (Cer). Cer is a membrane-bound molecule that is transported via vesicular transport or by the protein ceramide transfer protein (CERT) from the ER to the Golgi apparatus, where it acts as a precursor for the synthesis of other complex sphingolipids such as sphingomyelin (SM), GlcCer, etc. (Figure 1) [9]. Synthesis of GlcCer is an important first step for the synthesis of other GSLs and is catalyzed on the cytosolic side of the Golgi by the transmembrane enzyme GCS [9,10,11]. Once GlcCer is synthesized in the cytosol, it is translocated towards the Golgi lumen for the synthesis of other GSLs by multiple Golgi and Trans Golgi Network flippases [9,11,12,13]. Recently, it was shown that multiple ABC transporters differentially regulate GSL biosynthesis by acting as GlcCer flippases [14]. Furthermore, ABCC1 and ABCA12 have been shown to transport GlcCer in vitro [15] and in keratinocytes [16].

We have shown previously that ATP-binding cassette family A protein 1 (ABCA1) deficiency in humans and mice reduces plasma GlcCer levels [17]. However, induction, downregulation, inhibition, or knockdown of ABCA1 in human hepatoma Huh-7 cells had no effect on GlcCer efflux to HDL [17]. Recently, we identified that ABCC10 plays a role in the synthesis and efflux of GlcCer in Huh-7 cells [18], and ABCA1 may indirectly affect plasma GlcCer levels by playing a role in the biogenesis of HDL. We hypothesized that ABCC10 may act as a flippase of GlcCer and, therefore, regulate its synthesis and efflux. To test this possibility, we used different inhibitors of ABCC10 and studied their effects on the synthesis and efflux of GlcCer in Huh-7 cells.

## 2. Materials and Methods

### 2.1. Materials

C6-NBD ceramide (catalog #6224) was purchased from Setareh Biotech (Eugene, OR, USA). Tamoxifen (catalog #T5648) was purchased from Millipore Sigma (St. Louis, MO, USA). Cepharanthine (catalog #sc-39121) and sorafenib (catalog #sc-220125) were purchased from Santa Cruz (Dallas, TX, USA). All other chemicals and solvents were obtained from Fisher Scientific (Pittsburgh, PA, USA) or VWR International (Bridgeport, NJ, USA).

### 2.2. Mice

The ABCC10 knockout mice on C57BL6/J background [19] were a kind gift from Dr. Elizabeth Hopper-Borge of Fox Chase Cancer Center, Philadelphia, PA. Mice were housed under a 12:12 h light:dark schedule and bred at the animal facility in accordance with all requirements of KAIMRC IACUC. Mice had ad libitum access to chow diet and water.

### 2.3. Synthesis and Efflux of Sphingolipids

For efflux, Huh-7 cells were incubated with 2 µM C6-NBD ceramide in 10% FBS containing DMEM at 37 °C for 3 h, washed 3 times with DMEM plus 0.1% BSA, and the efflux was initiated by the addition of fresh DMEM containing 40 µg/mL of either BSA or human serum HDL (#MBS173147, MyBioSource, San Diego, CA, USA). Culture media were collected after 6 h of incubation and centrifuged (2500 rpm, 600× *g*, 15 min, 4 °C, Heraeus Fresco 21 Centrifuge, 75003424 rotor, ThermoFisher Scientific, Waltham, NJ, USA) to pellet detached cells. To study the effect of tamoxifen on GlcCer synthesis, Huh-7 cells were treated with different doses (0–10 µM) of tamoxifen for 17 h before the addition of C6-NBD ceramide to the cells. In subsequent experiments, Huh-7 cells were treated with 5 µM concentration of sorafenib or cepharanthine for 17 h before the addition of C6-NBD ceramide or during the efflux studies with HDL. Lipids in the media and cells were extracted [20], dried and re-suspended in 100 µL of isopropanol for the separation of sphingolipids on thin layer chromatograph (TLC) silica plates (#44931, Analtech, Inc., Newark, DE, USA) using a CHCl_3_:CH_3_OH:C_6_H_5_CH_3_:NH_4_OH:H_2_O (40:40:20:0.4:1.6, ratios by volume) solvent system [17,21]. The TLC plates were visualized with the ChemiDoc MP Imaging System from BioRad (Hercules, CA, USA), and bands corresponding to Cer, SM, and GlcCer were quantified using ImageJ software version 1.53t (NIH, Bethesda, MD, USA).

### 2.4. Cell-Free In Vitro GlcCer Synthesis Assay

Liver tissues from chow-fed *Abcc10^+/+^* and *Abcc10^−/−^* mice were homogenized in a buffer containing 50 mM Tris-HCl, 1 mM EDTA, 5% sucrose, pH 7.4 and a mixture of protease inhibitors. To study the effect of tamoxifen, a GlcCer synthesis assay was performed in liver homogenates from *Abcc10^+/+^* and *Abcc10^−/−^* mice in the presence of different doses (0–10 µM) of tamoxifen for 1 h. In a separate assay, liver homogenates isolated from wildtype mice were incubated with 5 µM concentration of ABCC10 inhibitors, sorafenib or cepharanthine. The homogenates (500 µg of protein) were added to assay buffer containing 50 mM Tris-HCl, pH 7.4, 25 mM KCl, C6-NBD ceramide (3.3 µg/mL), phosphatidylcholine (100 µg/mL), and UDP-glucose (500 µM) in a total volume of 200 µL reaction and incubated for 1 h at 37 °C [22,23]. The reaction was stopped by adding 200 µL of CHCl_3_/CH_3_OH (2:1, *v*/*v*). Lipids were extracted and subjected to TLC.

### 2.5. Subcellular Fractionation and Western Blotting

Livers (~200 mg) from wildtype mice were washed with ice-cold PBS and minced on ice using sharp scissors. Tissues were resuspended in 2 mL of homogenization buffer (0.25 M sucrose, 1 mM EDTA, 15 mM HEPES, pH 7.4) containing 1 mM PMSF and homogenized using Kontes Potter-Elvehjem homogenizer (DWK Life Sciences, Mainz Germany) with ~20 iterations of up and down passes of the pestle. Homogenate was centrifuged at 1000× *g* for 10 min at 4 °C (Heraeus Fresco 21 Centrifuge, 75003424 rotor, ThermoFisher Scientific, Waltham, NJ, USA). Supernatant was collected and centrifuged for another 60 min at 10,000× *g* at 4 °C. For density gradient separation, 2 mL of 80%, 60%, 40%, and 20% Percoll (#17-0891-01, GE Healthcare, Chicago, IL, USA) in HEPES/sucrose buffer (0.25 M sucrose, 15 mM HEPES, pH 7.4) were layered on top of a 1 mL cushion of saturated sucrose. The 10,000× *g* supernatant from the above tissue homogenate was diluted to 3 mL with the homogenization buffer and layered on top of the Percoll gradient solutions. Samples were ultracentrifuged at 284,000× *g* for 2 h at 4 °C (Himac CP100NX, P40ST rotor, Eppendorf Himac Technologies Co., Ltd., Ibaraki, Japan), and 500 µL fractions were collected from the top. Fractions were sonicated on ice using two 10 second pulses with a 30 second interval and applied to SDS-PAGE gel. Gels were blotted onto PVDF membrane and analyzed with ABCC10 (#ab230017, Abcam, Waltham, MA, USA) or the indicated housekeeper protein marker antibodies (Pan-Cadherin #4068; GM130 #12480; Calreticulin #12238; Vimentin #5741; GAPDH #5174, Cell Signaling Technology, Danvers, MA, USA). Proteins were visualized after applying specific secondary HRP-conjugated antibodies and exposure to Clarity Western ECL substrate (#1705060, BioRad, Hercules, CA, USA) by use of a ChemiDoc MP Imaging System from BioRad (Hercules, CA, USA).

### 2.6. Statistical Analysis

Data were plotted as replicates and were presented as the mean ± S.D. The mean values of each group were analyzed by Student’s *t* test using GraphPad Prism software (version 5.0; GraphPad, San Diego, CA, USA). The results with *p* < 0.05 were considered statistically significant.

## 3. Results

### 3.1. Tamoxifen Decreases GlcCer Synthesis and Efflux in Huh-7 Cells

**Figure 2:**
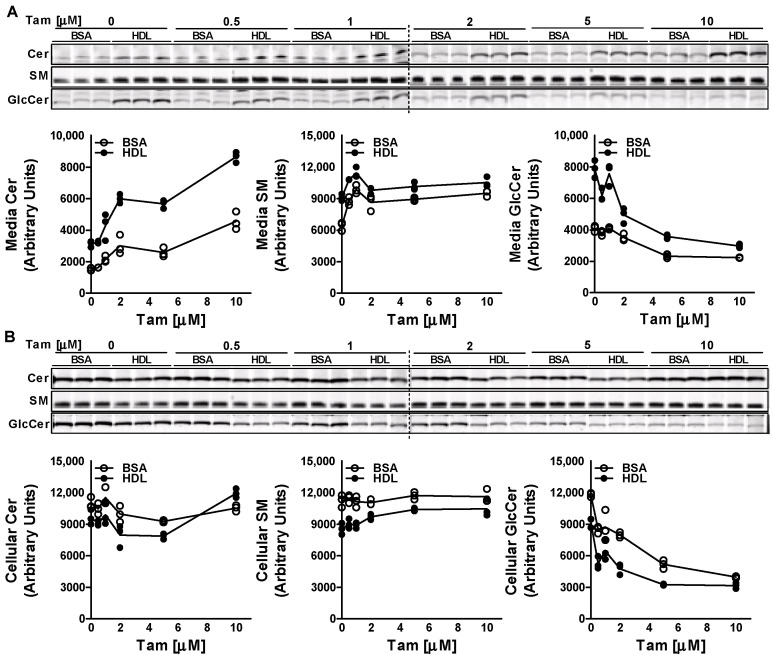
Effect of tamoxifen on the efflux and synthesis of GlcCer. Huh-7 cells were treated with different doses (0–10 µM) of tamoxifen (Tam) for 17 h, labeled with 2 µM of C6-NBD ceramides in 10% FBS containing DMEM for 3 h, washed three times with 0.1% BSA containing DMEM, and then incubated with either 40 µg/mL BSA or HDL for 6 h to study sphingolipid efflux. Lipids in the media (**A**) and cells (**B**) were extracted and separated on TLC plate, and fluorescence in Cer, SM, and GlcCer bands was visualized with the PhosphorImager and quantified using ImageJ software (plotted below each TLC image). Values are plotted as replicates.

Treatment of Huh-7 cells with increasing doses (0–10 µM) of tamoxifen for 17 h resulted in an increase in the efflux of Cer and SM to both BSA and HDL acceptors (Figure 2A). As expected, the increase in efflux of Cer and SM was higher in HDL-treated cells compared to that in BSA-treated cells at all doses tested. Interestingly, tamoxifen decreased the efflux of GlcCer to both BSA and HDL. These results suggest that tamoxifen differentially affects efflux of Cer, SM and GlcCer. Next, we measured the levels of sphingolipids in the cells. We did not detect much difference in the cellular levels of Cer and SM with the increasing doses of tamoxifen. However, compared to BSA-treated cells, levels of Cer and SM in HDL-treated cells were lower (Figure 2B). Treatment of Huh-7 cells with tamoxifen resulted in a dose-dependent decrease in the levels of GlcCer in the cells treated with BSA or HDL, suggesting that tamoxifen also decreases the synthesis of GlcCer in the cells.

### 3.2. Tamoxifen Does Not Affect Glucosylceramide Synthase Activity in Liver Homogenates

**Figure 3:**
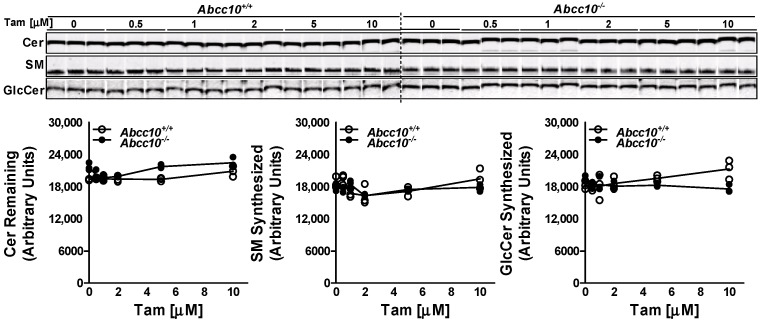
Tamoxifen does not affect GCS activity. Liver homogenates (500 µg protein) from *Abcc10^+/+^* and *Abcc10^−/−^* mice were incubated in triplicate with C6-NBD ceramides (3.3 µg/mL), phosphatidylcholine (100 µg/mL) and UDP-glucose (500 µM) in the presence of increasing concentrations of tamoxifen (0–10 µM) at 37 °C for 1 h. Lipids were extracted from the reaction mixture and separated on TLC plate. Fluorescence in Cer, SM, and GlcCer bands was visualized with the PhosphorImager (top) and quantified using ImageJ software (bottom). Values are plotted as replicates.

To rule out the possibility that tamoxifen may be inhibiting the GCS enzyme, we performed the cell-free assay in the liver homogenates isolated from *Abcc10^+/+^* and *Abcc10^−/−^* mice. Incubation of NBD-Cer with UDP-glucose and increasing doses of tamoxifen (0–10 µM) for 1 h with the liver homogenates from *Abcc10^+/+^* and *Abcc10^−/−^* mice did not show any significant difference in the synthesis of GlcCer or SM (Figure 3). Furthermore, we did not see any difference in the synthesis of GlcCer or SM in the liver homogenates isolated from the *Abcc10^+/+^* and *Abcc10^−/−^* mice. These combined data suggest that decrease in GlcCer levels in tamoxifen treated cells is not due to any reduction in GCS activity.

### 3.3. GlcCer Synthesis Is Decreased by ABCC10 Inhibitor Sorafenib but Not by Cepharanthine

**Figure 4:**
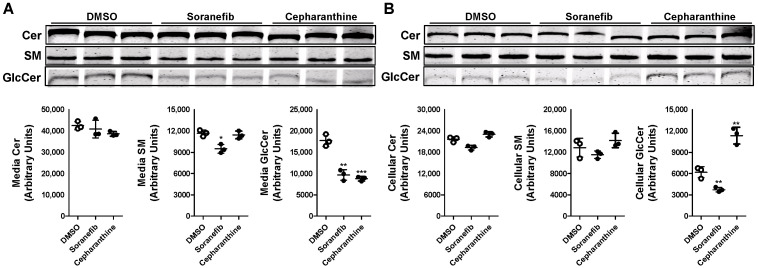
Pre-treatment of Huh-7 cells with ABCC10 inhibitors affects synthesis and efflux of GlcCer. Huh-7 cells were pre-treated with 5 µM concentration of sorafenib or cepharanthine for 17 h, labeled with 2 µM of C6-NBD ceramides in 10% FBS containing DMEM for another 3 h to study sphingolipid synthesis. Media were collected, and cells were washed three times with 0.1% BSA containing DMEM and then used to extract lipids. Sphingolipids were separated in the media (**A**) and cells (**B**) on TLC, and fluorescence in Cer, SM, and GlcCer bands was visualized with the PhosphorImager (top) and quantified using ImageJ software (bottom). Values are plotted as replicates (mean ± SD), * *p* < 0.05, ** *p* < 0.01 and *** *p* < 0.001 compared with DMSO-treated cells.

To confirm that the decrease in GlcCer synthesis with tamoxifen in Huh-7 cells involves ABCC10, we pre-treated Huh-7 cells with a 5 µM concentration of known ABCC10 inhibitors, sorafenib and cepharanthine [24] for 17 h. Next, we labeled the cells with C6-NBD ceramide for another 3 h in the presence or absence of these inhibitors to study the synthesis of sphingolipids. Media were also collected to measure the secretion of sphingolipids. We did not see any change in the levels of Cer in the media but saw a decrease of ~19% in the levels of SM in the sorafenib-treated cells (Figure 4A). There was also a significant reduction of around 45–50% in the levels of GlcCer in the media in sorafenib- and cepharanthine-treated cells. Next, we measured the amount of these sphingolipids in the cells. Treatment of Huh-7 cells with either sorafenib or cepharanthine did not result in any significant change in the cellular levels of Cer or SM (Figure 4B). However, we saw a reduction of ~40% in the levels of GlcCer when the cells were treated with sorafenib as compared to DMSO-treated cells (Figure 4B), suggesting that GlcCer synthesis is affected by sorafenib. Surprisingly, cepharanthine was unable to reduce the levels of GlcCer in the cells. On the contrary, we saw an increase of ~83% in the levels of GlcCer in cepharanthine-treated Huh-7 cells when compared to DMSO-treated cells (Figure 4B). These results suggest that cepharanthine affects only the secretion of GlcCer to the media, resulting in its accumulation in the cells.

**Figure 5:**
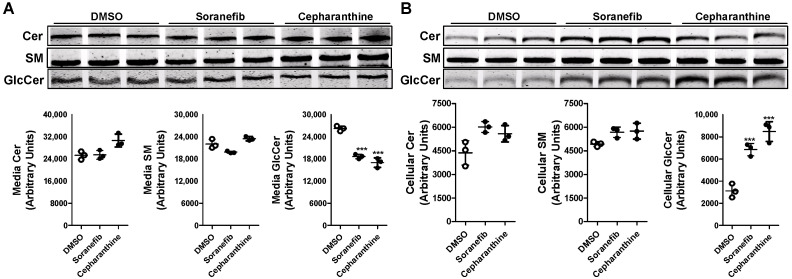
Post-treatment of ABCC10 inhibitors affects only the efflux and not synthesis of GlcCer in Huh-7 cells. Huh-7 cells were labeled with 2 µM of C6-NBD ceramides in 10% FBS containing DMEM for 3 h to synthesize HexCer. Cells were washed three times with 0.1% BSA containing DMEM, and then incubated with 40 µg/mL of HDL in the presence of 5 µM sorafenib or cepharanthine for 6 h to study GlcCer efflux. Media and cells were collected to extract lipids. Lipids were separated in the media (**A**) and cells (**B**) on TLC plate and fluorescence in Cer, SM, and GlcCer bands was visualized with the PhosphorImager (top) and quantified using ImageJ software (bottom). Values are plotted as replicates (mean ± SD), *** *p* < 0.001 compared with DMSO-treated cells.

The differential effect of sorafenib and cepharanthine on the levels of GlcCer in Huh-7 cells was intriguing. The data suggest that pre-treatment of Huh-7 cells with sorafenib decreases both the synthesis and efflux of GlcCer, whereas cepharanthine only inhibits the efflux. To rule out the effect of reduced sphingolipid synthesis on the efflux, we did not pre-treat Huh-7 cells with the inhibitors before or during the incubation with NBD-Cer to allow for the synthesis of GlcCer. Cells were treated with the 5 µM concentration of inhibitors only during the efflux of the sphingolipids in the media. There was a significant decrease of ~29–35% in the efflux of GlcCer in the media after the treatment of Huh-7 cells with both the inhibitors (Figure 5A). We also saw a significant increase of more than 2-fold in the accumulation of GlcCer levels in the cells after the treatment with both sorafenib and cepharanthine (Figure 5B). These data suggest that sorafenib and cepharanthine act as GlcCer efflux inhibitors in the cells. Furthermore, treatment of cells with sorafenib after the allowing for the synthesis of sphingolipids did not affect the synthesis of GlcCer.

**Figure 6:**
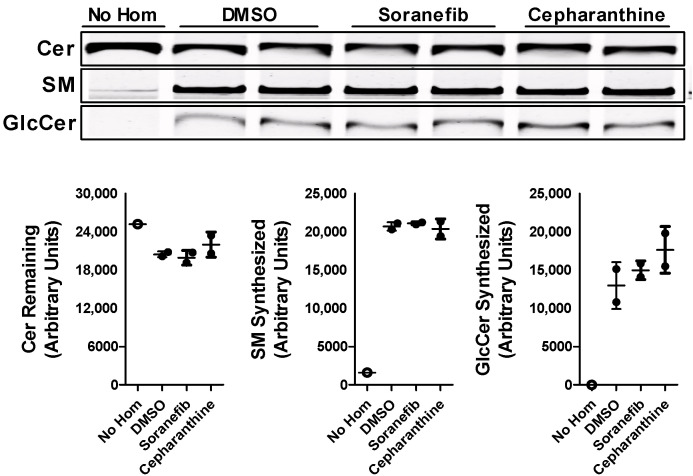
ABCC10 inhibitors does not affect GCS activity. Liver homogenates (500 µg protein) from wild type mice were incubated with C6-NBD ceramides (3.3 µg/mL), phosphatidylcholine (100 µg/mL) and UDP-glucose (500 µM) in the presence of 5 µM concentration of either sorafenib or cepharanthine at 37 °C for 1 h. Lipids were extracted from the reaction mixtures, separated on TLC, fluorescence in Cer, SM, and GlcCer bands were visualized with the PhosphorImager (top), and quantified using ImageJ software (bottom). Values are plotted as replicates (mean ± SD).

Changes in the levels of GlcCer in Huh-7 cells after the treatment with sorafenib or cepharanthine may be due to the changes in the activity of GCS. To test this possibility, we incubated liver homogenates isolated from wildtype mice with 5 µM concentration of either sorafenib or cepharanthine. In vitro cell-free assay showed that this change in GlcCer levels after the treatment with these inhibitors was not due to any change in the activity of GCS enzyme (Figure 6). These results suggest that sorafenib and cepharanthine are affecting GlcCer levels independent of GCS activity.

### 3.4. Localization of ABCC10 with Subcellular Membranes

**Figure 7:**
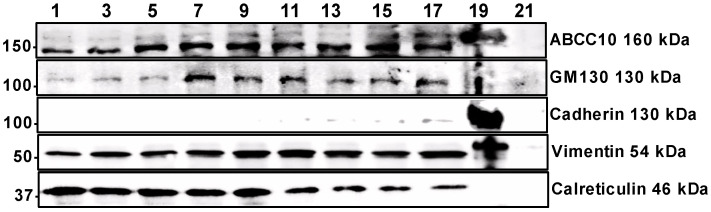
Localization of ABCC10 with subcellular membranes. Liver homogenates from wildtype mouse were used to separate the ER, Golgi and cell membranes using Percoll density gradient ultracentrifugation. Western blot analysis of subcellular fractions was performed using specific antibodies against ABCC10 or housekeeper protein markers of Golgi (GM1340), cell membrane (Cadherin), cytoskeleton (Vimentin), and ER (Calreticulin).

One of the plausible explanations for the differential effect of sorafenib and cepharanthine on the synthesis of GlcCer may be because these inhibitors are inhibiting ABCC10 at different subcellular localizations in the cells. To show that ABCC10 is localized at different subcellular membranes in the cells, we performed Percoll gradient subcellular fractionation of mouse liver homogenate to isolate different membranes and used housekeeper protein markers to identify these fractions (Figure 7). Our data showed that ABCC10 is expressed in cell membrane as well as other subcellular membranes such as ER and Golgi.

## 4. Discussion

We recently reported that ABCC10 plays an important role in the synthesis and secretion of GlcCer [18]. Budani et al. [14] showed that multiple ABC transporters potentially act as GlcCer flippases and differentially control GSL biosynthesis. Here, we showed that ABCC10 differentially regulates the synthesis and efflux of GlcCer in Huh-7 cells. We hypothesize that Golgi-bound ABCC10 may be involved in GlcCer synthesis, whereas cell membrane-bound ABCC10 may be involved in GlcCer efflux.

Several studies have shown that multi-drug resistant (MDR) cells exhibit abnormal elevation of the GlcCer level, which is reduced by tamoxifen treatment [25,26,27]. Studies have revealed that elevated levels of GlcCer in cancer cells correlate with overexpression of P-glycoprotein [25,28]. Tamoxifen has been shown to bind and antagonize P-glycoprotein [29,30,31]. It is now well-known that these cell lines express increased levels of ABCC10 that confer resistance to a variety of anticancer drugs [32,33,34]. These observations suggest that increased ABCC10 expression may also be involved in increased GlcCer levels in MDR cells. Our data are consistent with these published reports showing reduced GlcCer synthesis in the presence of tamoxifen. This decrease in GlcCer synthesis is not due to reduction in GCS enzyme activity, since cell-free in vitro assays in liver homogenates from *Abcc10^+/+^* and *Abcc10^−/−^* mice did not result in the inhibition of GCS. These results were again consistent with the published data showing that the synthesis of GlcCer was not inhibited by tamoxifen in the cell-free GCS assay using C6-ceramide as a substrate [31].

Sorafenib is a multi-kinase inhibitor [35] that inhibits the cellular efflux function of ABC transporters [36]. On the other hand, cepharanthine directly interacts with P-glycoprotein and inhibits its transport activity [37,38]. However, both sorafenib and cepharanthine have also been used to inhibit the ATPase activity of ABCC10 [24]. During prolonged pre-treatment of cells with sorafenib, we saw a reduction in both the cellular and media GlcCer levels. In contrast, prolonged pre-treatment with cepharanthine increased the levels of GlcCer in the cells and reduced the levels in the media. However, when cells were allowed to synthesize sphingolipids from NBD-Cer without the pre-treatment with these inhibitors, we observed that both sorafenib and cepharanthine, as expected, decreased the efflux of newly synthesized NBD-GlcCer to the media. Surprisingly, sorafenib, instead of decreasing the synthesis, increased the levels of GlcCer in the cells, similarly to cepharanthine. One plausible explanation for the differential effects of these two inhibitors on the synthesis and efflux of GlcCer may be due to the localization of ABCC10 on the cell plasma membrane and Golgi apparatus. Cell-free assays showed that the reduction in GlcCer levels in the cells was not due to inhibition of GCS enzyme activity. On the contrary, sorafenib treatment has been shown to increase the expression of both mRNA and protein levels of GCS in Hep3B and HepG2 cells [39]. These data suggest that changes in GlcCer levels in Huh-7 cells due to treatment with sorafenib or cepharanthine may be independent of the activity or protein levels of GCS enzyme. Studies have shown that GlcCer synthesis by GCS occurs on the cytoplasmic leaflet of the Golgi apparatus [11,40,41]. In this study, subcellular fractionation of liver cells showed that ABCC10 is located on both the cell membrane as well as subcellular membranes such as ER and Golgi (Figure 7).

**Figure 8:**
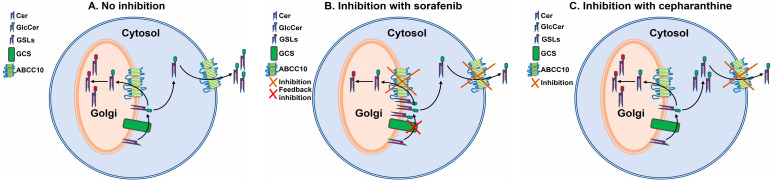
Proposed schematic representation of GlcCer transport by ABCC10 under different cellular stimuli. Under normal conditions, (**A**) glucosylceramide synthase (GCS) present on the cytosolic side of the Golgi converts ceramide (Cer) to glucosylceramide (GlcCer), which may be either translocated to the lumen by Golgi membrane-bound ABCC10 for further glycosylation to form complex glycosphingolipids (GSLs) or effluxed to the media by cell membrane-bound ABCC10. Inhibition of Golgi membrane- and cell membrane-bound ABCC10 with sorafenib (**B**) blocks translocation of GlcCer to the lumen or media and, therefore, may result in the feedback inhibition of GCS, leading to less synthesis of GlcCer. Inhibition of cell membrane-bound ABCC10 with cepharanthine (**C**) may prevent only the efflux of GlcCer to the media and, thereby, results in increased accumulation of GlcCer in the cells.

Under normal conditions, GlcCer synthesized on the cytosolic side of the Golgi must be translocated to the lumenal side for further GSL biosynthesis (Figure 8A). It is presumed that translocation of GlcCer from the cytosol to lumen is mediated by multiple Golgi and Trans Golgi Network flippases [9,11,12,13]. It is possible that ABCC10 present on the Golgi membrane may also act as one of the flippases. Some of the GlcCer may be effluxed out of the cells to the media by ABCC10 (Figure 8A). Down-regulation of ABCC10 with siRNA [18] or inhibition with sorafenib or tamoxifen may decrease the flippase activity of ABCC10 on the Golgi membrane, causing a feedback inhibition of GCS, leading to reduction in GlcCer synthesis in the cells, as has been shown with other transporter proteins such as ABCA3, ABCA4, ABCB10, ABCA12, and ABCB1 [14]. We have previously shown that microsomal triglyceride transfer protein (MTP) enhances cellular cholesteryl esterification by relieving the product inhibition of ACAT enzymes by transferring the products from the enzymes’ active site onto the apoB-containing lipoproteins [42]. In these studies, we showed that deletion or inhibition of MTP leads to accumulation of cholesteryl esters (products of ACAT enzymes) in the intestinal and liver cells, causing feedback inhibition of ACAT enzymes. Based on these studies and similarly to MTP, we presume that flippases may be important in removing GlcCer from the site of its synthesis on GCS enzyme and transferring it to the Golgi lumen. We speculate that any reduction in the activity of these flippases may lead to the feedback inhibition of the GCS enzyme and, thereby, reduce GlcCer synthesis in the cells. When cells are treated with sorafenib, it not only binds to the cell surface ABCC10, but due to its higher bioavailability (~39–48%) [43], it can enter the cells and bind to the subcellular membrane ABCC10 and, thereby, decrease both efflux of GlcCer to the media and translocation of GlcCer to the lumen of the Golgi, leading to less synthesis due to feedback inhibition of GCS (Figure 8B). On the other hand, it is possible that due to low compound bioavailability (~5%) [44], cepharanthine may fail to enter the cells and binds only the ABCC10 on the cell membrane to reduce the efflux of GlcCer to the media, consequently causing accumulation of GlcCer inside the cells (Figure 8C). Reduction in the synthesis of GlcCer in the presence of sorafenib and not cepharanthine suggests that Golgi membrane and not cell membrane ABCC10 may play a role in the synthesis of GlcCer.

In summary, we identified a novel role of ABCC10 in the biosynthesis and transport of GlcCer across the membranes. Our studies provide evidence that ABCC10 may act as a flippase that translocates GlcCer across the Golgi membrane.

We also observed that Golgi membrane-bound ABCC10 plays a role in the synthesis and transport of GlcCer that may be an important step for further glycosylation to synthesize other GSLs inside the Golgi lumen. Further studies are warranted to elucidate the exact mechanism of ABCC10 as flippase of GlcCer during the synthesis of GSLs.

## Data Availability

The data presented in this study are available on request from the corresponding author.
